# Hepatitis C virus and proprotein convertase subtilisin/kexin type 9: a detrimental interaction to increase viral infectivity and disrupt lipid metabolism

**DOI:** 10.1111/jcmm.13273

**Published:** 2017-07-18

**Authors:** Matteo Pirro, Vanessa Bianconi, Daniela Francisci, Elisabetta Schiaroli, Francesco Bagaglia, Amirhossein Sahebkar, Franco Baldelli

**Affiliations:** ^1^ Unit of Internal Medicine Department of Medicine University of Perugia Perugia Italy; ^2^ Unit of Infectious Diseases Department of Medicine University of Perugia Perugia Italy; ^3^ Biotechnology Research Center Mashhad University of Medical Sciences Mashhad Iran

**Keywords:** cholesterol, hepatitis C virus, lipids, lipoprotein, proprotein convertase subtilisin/kexin type 9

## Abstract

From viral binding to the hepatocyte surface to extracellular virion release, the replication cycle of the hepatitis C virus (HCV) intersects at various levels with lipid metabolism; this leads to a derangement of the lipid profile and to increased viral infectivity. Accumulating evidence supports the crucial regulatory role of proprotein convertase subtilisin/kexin type 9 (PCSK9) in lipoprotein metabolism. Notably, a complex interaction between HCV and PCSK9 has been documented. Indeed, either increased or reduced circulating PCSK9 levels have been observed in HCV patients; this discrepancy might be related to several confounders, including HCV genotype, human immunodeficiency virus (HIV) coinfection and the ambiguous HCV‐mediated influence on PCSK9 transcription factors. On the other hand, PCSK9 may itself influence HCV infectivity, inasmuch as the expression of different hepatocyte surface entry proteins and receptors is regulated by PCSK9. The aim of this review is to summarize the current evidence about the complex interaction between HCV and liver lipoprotein metabolism, with a specific focus on PCSK9. The underlying assumption of this review is that the interconnections between HCV and PCSK9 may be central to explain viral infectivity.

**Table:** 

• Introduction
• HCV life cycle
• HCV interaction with lipid and lipoprotein metabolism
– Lipids, lipoproteins and HCV structure
– Lipoprotein receptors and HCV entry into host cells
– Lipids and HCV replication, assembly and secretion
• PCSK9 and lipoprotein receptors
• The interaction between HCV and PCSK9
• Impact of anti‐PCSK9 mAbs on clinical outcome
• Conclusions

## Introduction

Hepatitis C virus (HCV) is a single‐stranded RNA virus with a particular tropism for the hepatocytes 1, 2. More than 185 million people (~3% of the world's population) are infected by HCV 3 with an incidence rate of HCV infection that is apparently decreasing in the last years 4. HCV infection, mostly in its chronic form, is an important cause of morbidity leading to cirrhosis, end‐stage liver disease and hepatocellular carcinoma 3.

From HCV interaction with the hepatocytes to virion particle release, viral life cycle comprises a series of events with a close virus–host interplay intersecting some milestones of liver lipid and lipoprotein metabolism, thereby leading to hypobetalipoproteinaemia and hypocholesterolaemia 5. Viral particle binding to the cell surface and subsequent internalization involve the interaction between HCV envelope molecules and some hepatocyte surface molecules, including different lipoprotein receptors 6, 7, 8. After fusion of the HCV envelope with the host cellular membranes, viral nucleocapsid uncoating allows RNA genome release and intracytoplasmic translation of the HCV polyprotein precursor 9. Then, HCV RNA replication occurs within subdomains of the endoplasmic reticulum (ER) (*i.e*. membranous web), the latter including both HCV non‐structural proteins and infected cell proteins and lipids. The assembly of newly replicated HCV RNA and structural proteins, which is mediated by both viral and host proteins, takes place in close connection with lipoprotein synthesis within lipid droplet (LD) platforms that are surrounded by the membranous web 9. In the ER and Golgi apparatus, viral envelope acquisition and maturation occur through a molecular pathway overlapping with lipoprotein secretion 9. The mature virions may be then released into circulation as lipoprotein‐like particles, named lipo‐viro‐particles (LVP), which contain different hepatocyte‐synthesized apolipoproteins 8, 10, 11, 12. It is believed that LVP particle‐associated apolipoproteins may increase viral infectivity, participate in lipoprotein receptor‐mediated viral entry and have a crucial role for intracellular maturation of HCV particles.

In recent years, the importance of PCSK9 in the regulation of liver lipoprotein metabolism has emerged 13, 14, 15. At least four hepatocyte surface proteins, which are involved in HCV entry [*i.e*. CD81, low‐density lipoprotein receptor (LDLR), very low‐density lipoprotein receptor (VLDLR) and scavenger receptor class B type 1 (SR‐B1)], are known to be modulated to some extent by PCSK9. Specifically, a concentration‐dependent impact of PCSK9 on liver cell CD81 expression has been documented 16, 17. Also, circulating PCSK9 down‐regulates LDLR expression on the hepatocyte surface, thus decreasing LDL catabolism and increasing plasma LDL‐cholesterol (LDL‐C) levels 18, 19. Although there is evidence that PCSK9 decreases VLDLR expression in adipose tissue 20, whether such a down‐regulation may occur in the liver is not established and the potential impact of PCSK9‐mediated change in VLDLR expression on HCV infection is still unknown. In addition, a significant down‐regulation of *SR‐B1* gene was induced by PCSK9 21, 22, although its impact on HCV life cycle and replication needs to be defined. Experimental and observational studies investigated whether some interaction between HCV infection and circulating plasma PCSK9 occurs, showing inconclusive results. Accordingly, both increased and reduced PCSK9 levels have been detected in HCV‐infected patients as compared with healthy individuals 23. In this regard, it is likely that HCV genotype and other confounders may explain such discrepancies. Further uncertainty is provided by the observation of the increased plasma PCSK9 levels in HCV patients that are coinfected with human immunodeficiency virus (HIV) 24. Hence, further investigation is warranted to explore this issue.

The purpose of this review is to discuss the current evidence about the tangled and complex interaction between HCV infection, lipoprotein metabolism and PCSK9 expression.

## HCV life cycle

HCV has been discovered in 1989 1; it is a positive‐sense, 9.6‐kilobase uncapped single‐stranded RNA virus of the Flaviviridae family, genus Hepacivirus, which is composed by a nucleocapsid (protein and genome) surrounded by a viral envelope (proteins and lipids). HCV genome contains a single open reading frame (ORF) flanked by 5′ and 3′ non‐translated regions (NTRs), encoding a polyprotein of about 3000 amino acids, depending on HCV genotype. After HCV polyprotein is synthesized, cleavage by viral‐ and host‐encoded proteases yields mature proteins, including structural proteins (*i.e*. core protein, E1 and E2 glycoproteins) and non‐structural proteins (p7 or NS1, p23 or NS2, p70 or NS3, p8 or NS4A, p27 or NS4B, p56/58 or NS5A, p68 or NS5B). The structural core protein, which forms the viral nucleocapsid, and the two envelope glycoproteins (E1 and E2), together with NS1 and NS2, are required for virus assembly, whereas the remainder non‐structural proteins are required for RNA replication and the HCV life cycle 2.

The HCV life cycle is cytoplasmic. A sequence of events that include particle binding to the cell surface, interactions with proteins at the intercellular junction and receptor‐mediated endocytosis facilitate virus entry into the hepatocytes. Specifically, circulating HCV particle envelope glycoproteins interact with the basolateral surface of hepatocytes after crossing the fenestrated endothelium of the liver sinusoids. Several hepatocyte surface molecules mediate HCV binding and internalization, including CD81, SR‐B1, the dendritic cell‐specific intercellular adhesion molecule‐3‐grabbing non‐integrin (DC‐SIGN or CD209) and the liver/lymph node‐specific intercellular adhesion molecule‐3 (ICAM‐3)‐grabbing integrin (L‐SIGN or CD209L), LDLR, the asialoglycoprotein receptor (ASGP‐R) and heparan sulphate proteoglycans (HSPGs). HSPG represents the first attachment site before the interaction of the virus with the other putative receptors 25, 26. Additional entry factors have been identified including claudin‐1 (CLDN1), occludin (OCLN), Niemann‐Pick C1‐like 1 (NPC1L1), transferrin receptor 1 (TfR1), epidermal growth factor receptor (EGFR) and VLDLR 7. After receptor‐mediated binding, clathrin‐mediated endocytosis and fusion of HCV with host cellular membranes are facilitated by specialized viral proteins. The fusion process and the viral nucleocapsid uncoating are then followed by the virus RNA release into the cytoplasm to serve as a mRNA for the HCV polyprotein precursor. Upon synthesis and cleavage of the polyprotein precursor, HCV non‐structural proteins participate in the formation of the membranous web, where RNA replication proceeds *via* a negative‐sense copy that serves as a template for the production of large amounts of positive‐sense RNAs. An important event in the HCV assembly is represented by the nucleocapsid formation, which is driven by the interaction between the HCV genome and viral structural proteins and is orchestrated by viral and host molecules. Specifically, newly synthetized core protein and replicated RNA are recruited to the ER in close proximity with LDs, where viral particle assembly occurs in a process that is tightly linked to lipoprotein synthesis. A lipid‐rich viral envelope, in which are anchored the envelope glycoproteins, is then acquired to surround the nucleocapsid by ER internal budding. Subsequently, HCV maturation procedes within the Golgi apparatus, resembling the VLDL secretion pathway 9.

## HCV interaction with lipid and lipoprotein metabolism

The HCV life cycle is closely linked to the metabolism of lipids and lipoproteins. Accordingly, HCV uses several host machineries involved in lipoprotein synthesis, maturation and degradation, thus acquiring a constellation of peculiar characteristics, which allow it to increase its infectiveness.

### Lipids, lipoproteins and HCV structure

The virion is made of a nucleocapsid surrounded by a lipid envelope embedded with glycoproteins E1 and E2 forming a highly glycosylated heterodimer. However, a variable fraction of circulating infectious HCV particles, the so‐called LVPs, may have a more complex lipoprotein‐like structure and composition. These LVPs contain apolipoproteins, including apolipoprotein (apo)B, apoCII, apoCIII and apoE, and high amounts of triglycerides, which may explain the low buoyant density for HCV LVPs (density below 1.06 g/ml) as compared with other viruses 27. Interestingly, both apoB100 (synthetized in the liver) and apoB48 (synthetized in the intestine) have been detected within LVPs, thus suggesting possible assembly and/or maturation of LVPs into the enterocytes other than into the hepatocytes 28. The proportion of LVP among the circulating viral particles varies among different HCV patients, and almost half of HCV RNA is detected in the LVP circulating plasma fraction.

Although the exact role of LVP lipids and apolipoproteins is still the subject of intense investigation, there is evidence that the interaction of serum lipoproteins with HCV might contribute to mask the virion from the action of neutralizing antibodies and to facilitate viral entry and secretion 10, 29. Also, lipoprotein receptor‐mediated HCV entry has been found to be dependent on the density of the LVPs 8. Finally, Boyer *et al*. 11 reported that the complex formed by HCV E1, E2, apoB and apoE may initiate LVP morphogenesis.

### Lipoprotein receptors and HCV entry into host cells

The entry of HCV into host cells involves different host factors. There is substantial evidence showing that HCV recognizes a target cell by binding to the mannose‐binding lectins L‐SIGN (mainly expressed on liver endothelium) and DC‐SIGN (expressed on dendritic cells).

E1 and E2 glycoproteins, which serve as the fusogenic subunits during the process of HCV entry, are believed to function as HCV capture receptors 12. In addition, E1 and E2 interact with the CD81 tetraspanin and lipoprotein receptors; this interaction allows the transfer of the virus from the surface to side gradually. Finally, tight junction proteins may help HCV entry by inducing clathrin‐mediated endocytosis 30.

Among lipoprotein receptors, SR‐B1 and LDLR are involved in the entry of HCV particles into host cells. Specifically, SR‐B1 is believed important but not essential for HCV entry into HuH‐7 cells, as the post‐infection intracellular HCV RNA levels in SR‐KO Huh7.5.1 cells were only slightly reduced compared to parental Huh7.5.1 cells [8]. In addition, SR‐B1 might be implicated in HCV LVP delipidation and the consequent E2 conformational changes, which expose the CD81‐binding site 31.

Similar to SR‐B1, also LDLR is considered as a possible entry factor for HCV. Syed *et al*. showed that HCV stimulated LDLR expression in HCV‐infected HuH‐7 cells and in liver tissue from patients with chronic hepatitis C 32. Albecka *et al*. demonstrated that a small interfering RNA targeting the LDLR in HuH‐7 cells reduced HCV infectivity; moreover, in the same study, a soluble form of the LDLR inhibited both HCV entry into the hepatocytes and its binding to the LDLR, suggesting a direct interaction between the HCV particle and the LDLR 6. Mazumdar *et al*. suggested that the association between HCV E1 and apolipoproteins might facilitate virus entry through LDLR 33. Despite that deficiencies of SR‐B1 and LDLR impaired HCV entry, there is evidence that SR‐B1 and LDLR have a redundant role in this process 8.

Ujino *et al*. demonstrated a novel HCV entry pathway involving an additional lipoprotein receptor 7. Under hypoxic conditions, HCV entry into host cells was increased by up‐regulating VLDLR expression and was independent of the CD81. In addition, in the same study it has been demonstrated that SR‐B1, LDLR, NPC1L1 and CLDN1 were not directly involved in VLDLR‐mediated HCV infection and that VLDLR‐mediated HCV entry required HCV E2 and apoE. Recently, it has been observed that exogenous expression of SR‐B1, LDLR and VLDLR rescued HCV entry in the SR‐B1 and LDLR double‐knockout cells, confirming that VLDLR has a role in HCV entry 8.

It is believed that apolipoproteins associated with HCV particles, especially apoE, participate in heparan sulphate‐, LDLR‐ and SR‐B1‐mediated HCV entry 34. Moreover, the release of core protein from infected cells was reduced, HCV infectivity was ablated and direct HCV cell‐to‐cell transmission was abrogated in the absence of apoE 35. However, the permissive role of apoE‐mediated HCV entry may not be always evident, as Prentoe *et al*. failed to find an effect of anti‐apoE antibody on SRB1‐ or LDLR‐dependent HCV entry 36. NPC1L1 is a receptor composed of 13 transmembrane domains and three large extracellular loops (*i.e*. LEL1, LEL2 and LEL3), which is involved in cholesterol homeostasis and absorption. A role of NPC1L1 in HCV infection has been recently proposed. Saniz *et al*. reported that incubation of HuH‐7 cells with specific antibodies directed to LEL's domains inhibited HCV infection only when LEL1 was blocked, whereas no inhibitory effect was observed when anti‐LEL2 and anti‐LEL3 antibodies were used. Hence, the authors suggested that HCV cell entry might depend on its ability to bind the NPC1L1‐LEL1 domain. In the same study, the same authors investigated if there was a relationship between the amount of cholesterol within the HCV particles and NPC1L1‐mediated HCV cell entry. Interestingly, cholesterol‐rich HCV particles (*i.e*. JFH^G451R^) exhibited an increased NPC1L1‐dependent cell entry, whereas cholesterol‐poor HCV particles (*i.e*. JFH pseudotype particles) exhibited NPC1L1‐independent cell entry 37. A recent study by Zhang *et al*. provided experimental evidence that genetic polymorphisms of the NPC1L1 gene were associated with HCV infection. Analysis of five single nucleotide polymorphisms (SNPs) of the NPC1L1 gene and of the associated haplotypes showed that the GCCTT haplotype was less frequent in HCV patients than in control patients whereas the opposite was found for the GCCCT haplotype. This result indicates that protection from HCV infection might derive from the first NPC1L1 haplotype (GCCTT), whereas an increased risk of HCV infection might be associated with the second haplotype (GCCCT) 38.

### Lipids and HCV replication, assembly and secretion

Lipoprotein metabolism is a highly regulated multistep process 39. Accumulating evidence suggests that HCV replication, assembly and secretion may divert liver lipoprotein metabolism for the virus requirements. HCV replication occurs in the membranous web that is in close connection with intracytoplasmic LDs, where viral assembly takes place. Modifications in lipid and protein composition of LDs have a variable impact on HCV production. Thus, inhibition of diacylglycerol acyltransferase‐1 (DGAT‐1), a key enzyme involved in triacylglycerol biosynthesis and LD maturation, influenced negatively HCV particle assembly 40. Inhibition of the protease subtilisin/kexin‐isozyme‐1/site 1 protease (SKI1/S1P), which plays a critical role in the activation of sterol regulatory element‐binding proteins (SREBPs) controlling cholesterol and fatty‐acid biosynthesis and LD composition, impaired HCV assembly 41. On the other hand, HCV infection induced the expression of the LD‐associated adipose differentiation‐related protein (ADRP), which is known to promote virion assembly and secretion 42.

ApoB, apoE and microsomal triglyceride transfer protein (MTP), which have a crucial role in the VLDL synthetic pathway, are closely linked with HCV assembly and secretion. A decreased production of HCV virion particles has been reported in apoB^−/−^ human hepatoma cells 43. HCV secretion was reduced significantly by silencing apoB mRNA 44. HCV production was reduced also by blocking VLDL assembly through knocking‐down or inhibition of MTP 45, 46. However, these observations were not always confirmed 47, 48, probably because of differences in VLDL synthesis capacity between different cell culture systems. It has been demonstrated that apoE interaction with HCV NS5A is crucial for the viral assembly process 49. Also, Lee *et al*. provided experimental evidence that apoE participated in the virion envelope acquisition and contributed to the quality control of secreted infectious viral particles 50. To confirm the impact of apoE on HCV life cycle, a specific small interfering RNA (siRNA)‐mediated knocking‐down of the apoE expression was associated with a significant suppression of HCV particle formation 47. Finally, HCV secretion is closely linked with the Golgi trafficking and secretory pathways of the infected hepatocyte. The interaction between the oxysterol binding protein (OSBP) and the viral non‐structural protein NS5A promotes HCV secretion. Accordingly, inhibition of OSBP phosphorylation influenced negatively HCV maturation and secretion 51, 52.

## PCSK9 and lipoprotein receptors

PCSK9 is a 692‐amino acid secreted glycoprotein that consists of a signal sequence followed by a prodomain, a catalytic domain and a cysteine‐ and histidine‐rich C‐terminal domain (CHRD). The structure of CHRD is organized in three Cys/His‐rich modules, termed M1, M2 and M3 53, 54. PCSK9 is synthesized as a zymogen and, in the ER, it undergoes autocatalytic cleavage, the latter process being required for PCSK9 maturation and secretion 55. Maturation and secretion are mediated by two proteins: Sec24A is required for the transport of PCSK9 from the ER to the Golgi 56 and sortilin that interacts with PCSK9 in the trans‐Golgi network 57. Current evidence supports the concept that PCSK9 has no substrate other than itself. The expression of PCSK9 is high in the liver, intestine, kidney and brain, and the secreted form is present in human plasma. The function of PCSK9 is the binding of specific cell surface receptors to escort them towards intracellular acidic endosome/lysosome degradation compartments 58, 59. Specifically, PCSK9 plays a key role in the regulation of hepatic LDLR function through receptor binding and targeting to intracellular lysosomal degradation 19; this leads to reduced LDLR recycling and expression on the cell membrane, decreased plasma LDL catabolism and increased plasma LDL‐C levels.

Loss‐of‐function mutations in *PCSK9* gene induce high hepatic levels of LDLR and increased plasma LDL‐C clearance, whereas gain‐of‐function *PCSK9* gene mutations and overexpression of recombinant PCSK9 reduce LDLR protein levels in the liver and cause severe hypercholesterolaemia 60, 61, 62. As an example, D374Y, a *PCSK9* gain‐of‐function mutation 63, causes a severe form of autosomal‐dominant hypercholesterolaemia by inducing a significantly increased degradation of the LDLR compared with the wild‐type (WT) protein.

The importance of PCSK9 in regulating LDLR fate and plasma LDL‐C levels is further supported by the results of several randomized controlled trials with anti‐PCSK9 monoclonal antibodies (mAbs), revealing an impressive reduction in plasma LDL‐C levels following their subcutaneous administration 64, 65, 66. An unexpected reduction in plasma lipoprotein(a) levels following anti‐PCSK9 mAb treatment has been documented 67; although the exact mechanisms regulating lipoprotein(a) catabolism are still unclear, increased LDLR expression has been proposed to explain plasma lipoprotein(a) level lowering after treatment with anti‐PCSK9 mAbs 68.

Besides the described functions of PCSK9, it has been shown recently that PCSK9 is able to modulate the levels of other proteins other than the LDLR 69. In this regard, an additional PCSK9 target includes the VLDLR, which is a trans‐membrane lipoprotein receptor of the LDLR family modulating the extra‐hepatic metabolism of triglyceride (TG)‐rich lipoproteins. Current evidence suggests the role of VLDLR in reelin signalling 70, tumour development, angiogenesis 71, fibrin‐mediated trans‐endothelial leucocyte migration, cholesterol uptake and neuronal migration in the developing brain 72. VLDLR is expressed by several tissues, including adipose tissue, heart, skeletal muscle, brain, and it is generally absent in the liver 73, 74. In humans, VLDLR consists of specific regions, including intracellular NPxY motif, O‐linked glycosylation sugar domain, YWTD or LDLR class B repeats (*i.e*. β‐propeller domain), epidermal growth factor (EGF)‐like repeats and cysteine class A repeats. This last domain, similar but not identical to that of other members of LDLR family, defines the different binding affinity of VLDLR and allows interaction with apoE‐containing particles 75.

Roubtsova *et al*. suggested that endogenous PCSK9 regulates VLDLR protein levels in adipose tissue. These authors showed that PCSK9^−/−^ mice had more cell surface VLDLR in perigonadal fat when compared with WT mice. This difference was even more significant when a gender‐separated analysis was performed; accordingly, male and female mice showed fourfold and 43‐fold more VLDLR than WT mice, respectively. In the same study, the LDLR^−/−^/PCSK9^−/−^ female mice had a 36‐fold increase in VLDLR expression. In addition, these authors demonstrated that adipose tissue VLDLR expression was regulated only by circulating but not local PCSK9 20.

Adorni *et al*. provided experimental evidence that PCSK9 can influence also SR‐B1, the latter receptor being involved in the HCV cell entry. Incubation of WT mouse peritoneal macrophages with PCSK9 induced a slight, but statistically significant down‐regulation of *SR‐B1* gene 21. Finally, Shen *et al*. suggested that PCSK9 contributed to subendothelial oxidized LDL accumulation, which in turn might reduce SR‐B1 expression 22.

## The interaction between HCV and PCSK9

**Figure 1:**
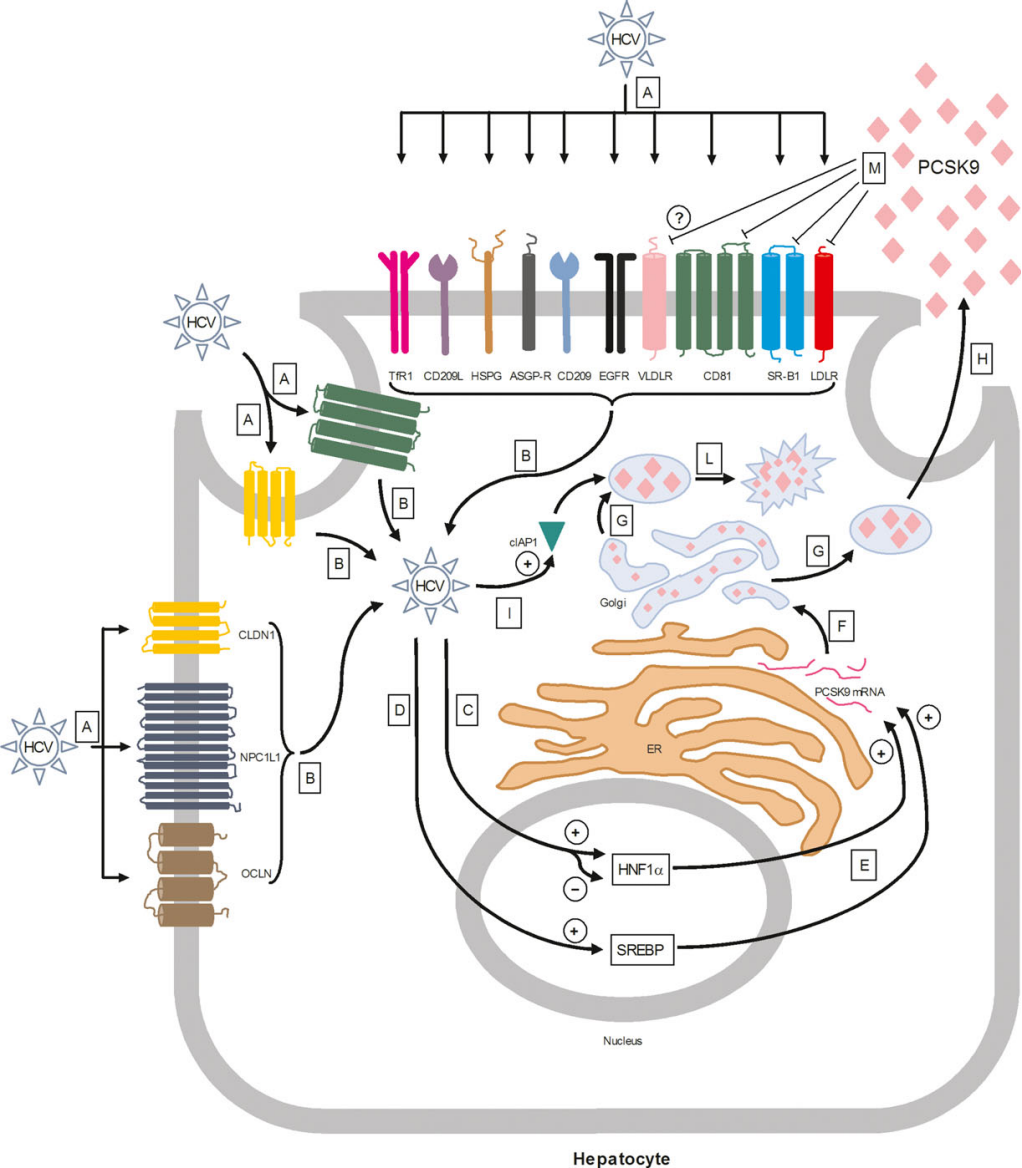
Key events in the interaction between HCV and PCSK9**.** (**A**) Interaction between HCV and surface receptors; (**B**) HCV intracellular entry; (**C**) HCV‐mediated HNF1α activation or inhibition; (**D**) HCV‐mediated SREBP activation; (**E**) SREBP‐ and HNF1α‐mediated PCSK9 mRNA stimulation; (**F**) PCSK9 protein maturation; (**G**) Formation of PCSK9‐secretory vesicles; (**H**) PCSK9 exocytosis; (**I**) HCV‐mediated cIAP1 activation; (**L**) cIAP1‐mediated PCSK9 proteasomal degradation; (**M**) PCSK9‐mediated CD81, SR‐B1, LDLR and VLDLR inhibition. HCV, hepatitis C virus; HNF1α, hepatic nuclear factor 1 alpha; SREBP, sterol regulatory element‐binding protein; PCSK9, proprotein convertase subtilisin/kexin type 9; cIAP1, cellular inhibitor of apoptosis protein 1; SR‐B1, scavenger receptor class B type I; LDLR, low‐density lipoprotein receptor; VLDLR, very low‐density lipoprotein receptor.

Several surface proteins (CD81, CLDN1, OCLN) and lipoprotein receptors (LDLR, VLDLR, SR‐B1) have been suggested to play a role in HCV infection. Some of these proteins and receptors, such as CD81, LDLR, VLDLR and SR‐B1, are regulated by PCSK9 (Fig. 1).

Labonte *et al*. provided *in vitro* experimental evidence that PCSK9, at supra‐physiological concentrations (7 μg/ml), reduced cell surface expression of LDLR and CD81 in HuH‐7 cells, thereby impeding HCV infection. Furthermore, PCSK9‐mediated CD81 inhibition was independent of LDLR expression 16. However, it should be underlined that the same effect was not observed when physiological concentrations of PCSK9 have been used in the study by Ramananathan *et al*. 17. In the referred study, extracellular PCSK9 regulated only LDLR, but there was no functional relationship between the amount of the secreted or soluble form of PCSK9 and CD81 levels. To confirm the latter result, *in vitro* treatment of HuH‐7 cells with alirocumab, a fully human anti‐PCSK9 mAb, reduced PCSK9 but had no impact on CD81 levels and neither on HCV entry and replication into hepatocytes. Hence, it appears that PCSK9 influences LDLR in HuH‐7 cells, but the impact on CD81 expression is dependent on PCSK9 concentrations. PCSK9 is also able to down‐regulate VLDLR and SR‐B1 expression. However, whether PCSK9‐mediated down‐regulation of the VLDLR occurs in the liver has not been defined; in addition, the potential impact of PCSK9‐mediated change in VLDLR and SR‐B1 expression on HCV infection is still unclear.

Important apolipoproteins and enzymes involved in lipoprotein synthesis and also in HCV intracellular assembly, namely apoB and MTP, are modulated by PCSK9. In particular, PCSK9 has a function in facilitating the production of liver apoB100 and intestinal apoB48 lipoproteins 76, 77. Also, it has been demonstrated that MTP protein levels and lipid transfer activity were enhanced in enterocytes by PCSK9, whereas PCSK9 siRNA inhibition lowered MTP protein levels and activity 77. Also DGAT‐1 is involved in HCV assembly 40. In the study by Rashid *et al*. 77, PCSK9 was able to increase enterocyte DGAT‐2 expression without affecting that of DGAT‐1. It must be emphasized, however, that the latter result was obtained in cultures of enterocytes. Overall, these data may be of interest for a better understanding of the impact of PCSK9 on key apolipoproteins and enzymes involved in HCV assembly (Table 1 **).** Nevertheless, evidence showing that PCSK9 modulation might impact on HCV assembly through a direct interference with these apolipoproteins and enzymes is still lacking.

**Table 1:** PCSK9‐modulated factors relevant to HCV life cycle

Factor	Effect	Method	Model	Reference
LDLR	↓protein expression	Treatment with supra‐physiological concentration of purified soluble PCSK9. Tested doses 0–15 μg/ml Effective dose >7 μg/ml	HuH‐7 human hepatocyte‐derived carcinoma cells	16
	↓protein expression	Treatment with physiological concentration of human recombinant PCSK9. Tested doses 5–500 nM Effective dose >5 nM	HuH‐7 human hepatocyte‐derived carcinoma cells	17
	↓protein expression	Treatment with human recombinant PCSK9. Tested doses 0.5–2.5 μg/ml Effective dose >0.5 μg/ml	HepG2 human hepatocyte‐derived carcinoma cells	18
CD81	↓protein expression	Treatment with supra‐physiological concentration of purified soluble PCSK9. Tested doses 0–15 μg/ml Effective dose >7 μg/ml	HuH‐7 human hepatocyte‐derived carcinoma cells	16
	no effect	Treatment with physiological concentration of human recombinant PCSK9. Tested doses 5–500 nM Effective dose >5 nM		17
VLDLR	↓protein expression	Induction of PCSK9 expression in Pcsk9^−/−^ mice.	C57BL/6J mouse	20
SR‐B1	↓mRNA expression	Treatment with human recombinant PCSK9. Tested dose 6.4 μg/ml	C57BL/6 mouse peritoneal macrophages	21
DGAT1	no effect	Treatment with human recombinant PCSK9. Tested doses 0–12.5 μg/ml Effective dose >10 μg/ml	CaCO‐2 human enterocytes	77
DGAT2	↑mRNA expression	Treatment with human recombinant PCSK9. Tested doses 0–12.5 μg/ml Effective dose >10 μg/ml	CaCO‐2 human enterocytes	77
MTP	↑mRNA expression	Treatment with human recombinant PCSK9. Tested doses 0–12.5 μg/ml Effective dose >10 μg/ml	CaCO‐2 human enterocytes	77
Apo B100	↑protein production	Treatment with human recombinant PCSK9. Tested doses 0–12.5 μg/ml Effective dose >10 μg/ml	CaCO‐2 human enterocytes	77
Apo B48	↑protein production	Treatment with human recombinant PCSK9. Tested doses 0–12.5 μg/ml Effective dose >10 μg/ml	CaCO‐2 human enterocytes	77

ApoB, apolipoprotein B; DGAT, diacylglycerol acyltransferase; LDLR, low‐density lipoprotein receptor; MTP, microsomal triglyceride transfer protein; PCSK9, proprotein convertase subtilisin/kexin type 9; RNA, ribonucleic acid; SR‐B1, scavenger receptor class B type 1; VLDLR, very low‐density lipoprotein receptor.

Contrary to what has been observed in healthy individuals, Bridge *et al*. showed that plasma PCSK9 did not correlate with plasma LDL‐C and total cholesterol levels in HCV‐infected patients, thus suggesting that HCV induces disruption of lipid homeostasis. Moreover, in the same study, plasma PCSK9 levels in HCV genotype 3 (HCV‐G3) and HCV‐G1 patients were lower and higher than healthy non‐HCV patients, respectively. In agreement, while high LVP particle levels in HCV‐G3 patients were associated with lower PCSK9 levels, the opposite was observed in HCV‐G1 patients, in which high LVP particle levels were associated with higher PCSK9 levels 23. Thus, also HCV genotype appears to be crucial in modulating PCSK9 levels. However, it must be emphasized that the results of this study were derived from a small‐size sample of HCV patients; hence, further research is warranted to confirm this observation. A recent study by Kohli *et al*. 24 add further uncertainty on the relationship between HCV infection and plasma PCSK9 levels. Accordingly, HCV infection was associated with a significant increase in plasma PCSK9 levels among HIV coinfected patients. HCV genotype was not available and reported in this study; hence, whether the increased plasma PCSK9 levels observed in HIV‐HCV coinfected patients might be the consequence of a greater prevalence of HCV‐G1 patients is unknown.

PCSK9 is a direct transcriptional target of SREBPs 78 and it is well known that HCV stimulates SREBPs activation 79, 80. Accordingly, SREBP activation is able to induce PCSK9 gene transcription along with hepatocyte nuclear factor 1α (HNF1α) 81 (Fig. 1). Hence, it would be conceivable that exposure to HCV would increase PCSK9 mRNA and protein levels. However, Syed *et al*. reported that HCV down‐regulates PCSK9 protein expression without affecting to a significant extent mRNA levels 32. Although the exact mechanism explaining such a result is still unclear, it must be underlined that both a down‐regulation and up‐regulation of HNF1α have been described after HCV infection 82, 83. Thus, the differential impact of HCV on SREBP and HNF1α might explain at least in part the lack of a significant PCSK9 mRNA level increase during HCV infection 32. To explain the reduced PCSK9 protein level following HCV infection, it has been observed that HCV induced PCSK9 proteosomal degradation by regulating the expression of the cellular inhibitor of apoptosis protein 1 (cIAP1) 84. Also, in cell lysates derived from HuH‐7 cells, Western blot analysis showed that HCV‐JC1 genotype 2a infection induced a modest increase in cIAP1 protein 32.

Blanchet *et al*. formulated another hypothesis on the regulation of PCSK9 levels induced by HCV, which is based on the key protein SKI‐1/S1P modulation. The authors showed that inhibition of SKI‐1/S1P, a lipogenic pathway regulator activating SREBP, impaired HCV genome replication and virion secretion 85. Also, Olmstead *et al*. 41 demonstrated that SKI‐1/S1P inhibition blocked HCV infection in hepatoma cells. Interestingly, SKI‐1/S1P inhibition induced also a strong reduction in PCSK9 mRNA and protein levels, which would be expected to increase HCV infectivity because of greater receptor‐mediated virus entry. However, in this study PCSK9 level reduction following SKI‐1/S1P inhibition was not associated with an increased CD81 expression and a decreased LDLR and NPC1L1 expression has been documented 86. Hence, additional antiviral SREBP‐independent actions of SKI‐1/S1P inhibition have been suggested to explain such discrepancies.

Additional treatments affecting either HCV life cycle or PCSK9 levels might provide novel insight for the understanding of the link between HCV infection and PCSK9 pathway. In this regard, a recent study by Blanchet *et al*. 87 found that statin treatment might have proviral effects by increasing LDLR and NPC1L1 expression. Conversely, high statin doses were associated with a net antiviral effect that was attributed to the down‐regulation of CLDN1 expression. Also, the statin‐mediated plasma PCSK9 level increase, which is associated with an increased surface receptor degradation, might further support the possible antiviral effect of statin therapy. Finally, a recent study by Hyrina *et al*. 88 found that plasma PCSK9 concentrations were up‐regulated in HCV patients who achieved a sustained virologic response following antiviral therapy. This study further supports the ability of HCV to usurp host‐cell metabolic pathways to increase its intracellular entry through PCSK9‐controlled surface receptors.

PCSK9 inhibition is currently considered an effective strategy for lowering plasma LDL‐C levels 64, 65, but its impact on HCV infection has not been explored so far in clinical trials. Hence, whether approved anti‐PCSK9 mAbs, by reducing LDLR degradation, may increase infectivity in HCV‐positive patients is unknown. At least three lines of evidence should be considered in this regard. First, as already mentioned, increased availability of hepatocyte LDLR during anti‐PCSK9 therapy might increase HCV‐LDLR interaction and cell entry. Second, PCSK9^−/−^ mice undergoing experimental liver resection exhibited reduced liver regenerative capacity and necrotic lesions, which were prevented by a high‐cholesterol diet 89. Thus, anti‐PCSK9 treatment might in theory increase the risk of HCV infectivity and reduce liver regeneration following hepatic injury. However, a preliminary third line of evidence seems to attenuate this fear as inhibition of PCSK9 with alirocumab did not result in increased susceptibility to HCV entry *in vitro* 17.

Nonetheless, concern about the potential unfavourable impact of PCSK9 inhibition on HCV infection progression has not been supported by the occurrence or progression of other infectious diseases. Whether PCSK9 and PCSK9 inhibition may alter the life cycle and activity of other viruses including hepatitis B virus (HBV) and HIV has not been established yet. Specifically, concern on the use of PCSK9 inhibitors in HCV‐infected patients cannot be easily translated to HIV patients, where cholesterol‐lowering treatment is often indicated (e.g. statins) and in some cases hampered by drug‐to‐drug interactions 90. Conversely, preclinical and clinical studies showed that PCSK9 inhibition promotes pathogen lipid clearance by LDLR, reduces inflammatory signals and improves prognosis among patients with sepsis 15, 91.

## Impact of anti‐PCSK9 mAbs on clinical outcome

Recently, Food and Drug Administration approved the clinical use of two anti‐PCSK9 mAbs: evolocumab and alirocumab. Currently, PCSK9 mAbs represent an additional lipid‐lowering therapeutic option either for patients with familial hypercholesterolaemia, those at very high cardiovascular risk and those who are intolerant to statin therapy. The use of these mAbs is particularly useful when current cholesterol‐lowering treatments do not allow to reach the recommended therapeutic LDL‐C goals. Thus, for instance, alirocumab reduced LDL‐C by 45% in patients with statin intolerance, as compared with a 14.6% reduction in LDL‐C with ezetimibe, and reduced significantly skeletal muscle adverse events as compared with atorvastatin 92. Also, evolocumab reduced LDL‐C by 56% in patients with statin intolerance as compared with a 36% reduction with ezetimibe 93. In patients with familial hypercholesterolaemia, alirocumab reduced LDL‐C by 48.8%, and evolocumab by 59.2% (every 2 week dose) and 61.3% (monthly dose) 94, 95. In the post hoc analysis of the ODYSSEY LONG TERM study, alirocumab reduced the combined end‐point of death from coronary artery disease, non‐fatal myocardial infarction, fatal or non‐fatal ischaemic stroke or unstable angina requiring hospitalization, as compared with placebo in patients with hypercholesterolaemia on maximally tolerated statin therapy 96. The ongoing ODYSSEY Outcomes trial (ClinicalTrials.gov Identifier: NCT01663402) will establish the long‐term effects of alirocumab on cardiovascular disease in 18,000 patients on maximally tolerated statin therapy. In the OSLER 1 and 2 trials, enrolling patients who completed one of the phase 2 or phase 3 studies of evolocumab, composite adverse cardiovascular events were significantly lower in patients receiving evolocumab as compared with standard therapy 97, 98. In the FOURIER trial, evolocumab on top of statin therapy reduced significantly the risk of cardiovascular events 99. Overall, the results from the above‐mentioned studies show that anti‐PCSK9 mAbs are potent and well‐tolerated cholesterol‐lowering drugs, with a significant clinical benefit on cardiovascular outcome. Despite that the use of these drugs is actually a novel and promising therapeutic tool for cardiovascular prevention, clinical evidence supporting their use in HCV‐infected patients is still lacking. However, in the light of the dramatic rate of HCV eradication with highly active antiviral agents, whether anti‐PCSK9 treatment might become a valuable treatment option for cardiovascular prevention also in these patients needs to be established.

Irrespective of the possibility of using PCSK9 inhibitors in HCV‐infected patients, there are other cholesterol‐lowering options to be used in HCV patients. Despite caution is needed when prescribing statins to patients with advanced or acute liver disease, different lines of evidence support their use in HCV‐infected patients. First, statins may contribute to the clearance of HCV from the blood 100. Second, statin use has been associated with a dose‐dependent reduction in the risk of chronic liver disease progression to cirrhosis and hepatocellular carcinoma 100. Third, statin use decreased the decompensation rate in both HBV‐ and HCV‐related cirrhosis 101. Finally, statins ameliorated different measures of vascular injury and decreased the risk of cardiovascular events when compared with placebo in a large number of clinical settings 102, 103, 104, although direct evidence of statin‐induced cardiovascular prevention in HCV‐positive patients is not available. The use of ezetimibe would be also an option for cholesterol‐lowering in HCV patients. As ezetimibe is able to reduced plasma cholesterol levels and cardiovascular risk 105 by inhibiting intestinal NPC1L1 transporter, its use may be considered of particular therapeutic value also in patients with HCV infection. Intriguingly, HCV cell entry might depend on its ability to bind the NPC1L1‐LEL1 domain, thus making ezetimibe use in HCV‐positive patients a suitable option for these patients. Notably, a recent pilot study on two patients showed that ezetimibe was associated with a transient reduction in HCV viral load after liver transplantation 106. Whether nutraceuticals and novel drugs targeting both lipid metabolism and inflammation might provide an additional effective and safe therapeutic option to obtain a significant reduction in cardiovascular risk in HCV‐infected patients needs to be established 66, 107, 108.

## Conclusions

HCV life cycle is a clear example of the impressive plasticity of this Flavivirus. Accordingly, HCV is able to hijack endogenous lipid metabolic pathways for its own requirements, from virus–host interaction, to intracellular entry, replication and maturation up to extracellular secretion. PCSK9 is an ubiquitously expressed enzyme whose more studied function is represented by its ability to direct LDLR towards intracellular degradation, thus regulating plasma and intracellular cholesterol levels. A complex link between HCV infection and PCSK9 has emerged from the literature, in which a bidirectional loop of interactions is conceivable. Accordingly, PCSK9 has been involved in the regulation of key HCV entry receptors, including LDLR, VLDLR, SR‐B1 and CD81, thereby impeding in some cases of HCV infection. Also, PCSK9 is able to influence key factors of the lipid metabolism that are also involved in the HCV assembly, namely apoB and MTP. On the other hand, HCV was found to regulate PCSK9 expression by modulating its levels at a translational but not at a transcriptional level. Thus, HCV‐mediated proteasomal PCSK9 degradation has been described, along with SREBP activation and both inhibition and activation of the potent PCSK9 transcription factor activator HNF1α. Overall, current evidence seems to reinforce the concept that HCV dysregulates cell metabolism to promote the surface expression of membrane receptors that are in most cases under PCSK9 control. The complexity of the interaction between HCV, PCSK9 and lipid metabolism is further supported by the observation that the classical positive association between PCSK9 and LDL‐C levels that is observed in healthy individuals is lost in HCV patients. Additional research is therefore awaited to further characterize the intricate connections between HCV and PCSK9, and to provide a rationale for the correct use of current treatments targeting PCSK9 either directly or indirectly.

## Conflicts of interest

The authors confirm that there are no conflicts of interest.
